# Aflatoxin Contamination Detected in Nutrient and Anti-Oxidant Rich Edible Stink Bug Stored in Recycled Grain Containers

**DOI:** 10.1371/journal.pone.0145914

**Published:** 2016-01-05

**Authors:** Robert Musundire, Isaac M. Osuga, Xavier Cheseto, Janet Irungu, Baldwyn Torto

**Affiliations:** 1 International Centre of Insect Physiology and Ecology (*icipe*), Behavioural and Chemical Ecology Department, P.O. Box 30772–00100, Nairobi, Kenya; 2 Department of Crop Science and Postharvest Technology, Chinhoyi University of Technology, Off Chirundu Road, Bag 7724, Chinhoyi, Zimbabwe; 3 Department of Agricultural Resources Management, Kenyatta University, P.O. Box 43844–00100, Nairobi, Kenya; United States Department of Agriculture, Beltsville Agricultural Research Center, UNITED STATES

## Abstract

Recently, there has been multi-agency promotion of entomophagy as an environmentally-friendly source of food for the ever increasing human population especially in the developing countries. However, food quality and safety concerns must first be addressed in this context. We addressed these concerns in the present study using the edible stink bug *Encosternum delegorguei*, which is widely consumed in southern Africa. We analysed for mycotoxins, and health beneficials including antioxidants, amino acids and essential fatty acids using liquid chromatography coupled to quadrupole time of flight mass spectrometry (LC-Qtof-MS) and coupled gas chromatography (GC)-MS. We also performed proximate analysis to determine nutritional components. We identified the human carcinogen mycotoxin (aflatoxin B_1_) at low levels in edible stink bugs that were stored in traditonally woven wooden dung smeared baskets and gunny bags previously used to store cereals. However, it was absent in insects stored in clean zip lock bags. On the other hand, we identified 10 fatty acids, of which 7 are considered essential fatty acids for human nutrition and health; 4 flavonoids and 12 amino acids of which two are considered the most limiting amino acids in cereal based diets. The edible stink bug also contained high crude protein and fats but was a poor source of minerals, except for phosphorus which was found in relatively high levels. Our results show that the edible stink bug is a nutrient- and antioxidant-rich source of food and health benefits for human consumption. As such, use of better handling and storage methods can help eliminate contamination of the edible stink bug with the carcinogen aflatoxin and ensure its safety as human food.

## Introduction

To meet the growing needs of the world population, currently at 2.3% per year in Sub-Saharan Africa [1], there is need to re-evaluate the source of food and how it is produced. The Food and Agricultural Organization of the United Nations (FAO) proposed a global initiative to increase use of insects as food and feed in order to augment the current efforts to ensure future food security [2]. This initiative was based on the fact that insects are consumed as food (Entomophagy) by various ethnic groups in several countries in Central and South America, Oceania, Asia and Africa [2]. They have been shown to have vital nutritional components such as proteins, amino acids, fats, carbohydrates, vitamins and trace elements [2,3]. Additionally, they are also reported to contain important phytosterols for human health such as β-sitosterol, campesterol and stigmasterol [4].

The edible stink bug, *Encosternum delegorguei* Spinola (Hemiptera: Tessaratomidae) is widely distributed in subtropical woodland and bushveld in Zimbabwe and Northern provinces of South Africa [5, 6]. This bug plays an important role towards attainment of food and nutritional security as well as a source of income to rural communities [7, 8,9]. It is a phytophagus insect and feeds on sub-tropical savannah plants such as *Uapaca kirkiana* Müll. Arg. (Phyllanthaceae) and *Oxyanthus speciosus* DC (Rubiaceae) [6], *Dodonaea viscosa* Jacq. var. *angustifolia* (L.f.) Benth. (Sapindaceae), *Diospyros mespiliformis* Hochst. ex A.DC (Ebenaceae) [2,10], *Vangueria apiculata* (Verdc.) Lantz (Rubiaceae) among others. Its egg laying sites include grass stems and twigs of *Combretum imberbe* Wawra (Combretaceae) and *Combretum apiculatum* Sond. (Combretaceae) [11]. Host plant associations have potential implications on the chemical composition of the insects and consequently on their nutritional status.

**Fig 1:**
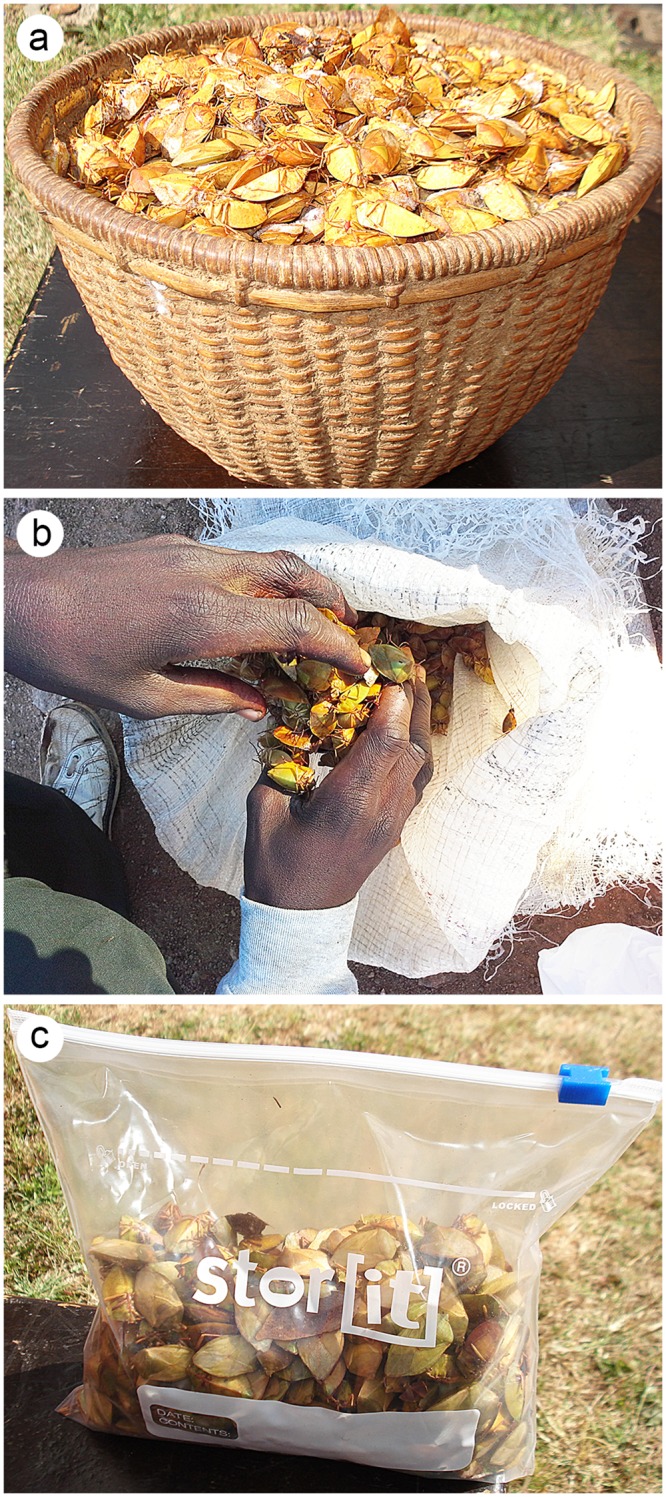
Method of storage of processed insects a) traditionally wooven wooden baskets, b) used grain bags and c) clean zip lock bags.

In Zimbabwe, harvesting of *E*. *delegorguei* is previously described [11] however, with modifications. Briefly, insects are collected from tree branches in the early hours of the day using the knockdown approach with the aid of long hooks or by climbing trees and vigorously shaking off insects perched on branches and leaves. The collected insects are then processed for consumption using a warm water killing and heating procedure before being stored in traditionally woven wooden baskets or in used grain bags (Fig 1) for later consumption or sale. These handling and storage practices are likely to have an impact on the nutritional status and food safety quality attributes of the bugs. The relationships between traditional harvesting; processing practices and nutrition; incidences of mycotoxins contamination in the edible stink bugs have not been studied.

We hypothesised that traditional harvesting and storage practices of *E*. *delegorguei* favour the occurrence of mycotoxins. Our study therefore sought to (i) investigate the effect of traditional handling and storage of the edible stink bug on mycotoxin contaminations with emphasis on aflatoxins, and (ii) profile the nutritional and phytochemical composition of the edible stink bugs in relation to processing methods used in South-eastern districts of Zimbabwe.

## Materials and Methods

### E. delegorguei

Samples of *E*. *delegorguei* were collected from Jiri Forest (approximately 20°2' 55" S 31° 43' 36" E) in Nerumedzo area of Bikita district, South-eastern Zimbabwe during the peak harvesting period (June, 2014) as this time of the year (May-August) consides with highest abdominal fat composition in *E*. *delegorguei* in South Africa Dzerefos et al. [11]. The forest is managed by the traditional customs under a village head who oversees forest conservation and harvesting activities of the edible stink bug. Harvesting of the bugs for this study was therefore done with the permission of the village head. The edible stink bug is utilized by the local community from the forest and therefore, this study did not involve endangered or protected species or endanger other wildlife in the forest.

Four harvesting quadrants (100 m^2^) were demarcated equidistant from each other and ensuring coverage of the entire forest (approximately 8 km^2^). Harvesting in each quadrant was carried out according to two procedures from branches of 10 randomly selected trees. In one approach, 5 kg of the insects were collected from each quadrant into wooden baskets that were sourced locally and then transfered into perforated re-used polypropylene grain bags (traditional practice) [11,12]. The other procedure involved harvesting insects directly into perforated clean zip lock bags. In both instances, perforation of bags allowed air circulation thus keeping the insects alive as per traditional practices until processing.

The harvested insects were then pooled to give one composite sample (20 kg) for each respective sampling procedure. This was based on the observation that traditional harvesting practices allow unrestricted harvesting of the edible stink bugs from as many parts of the forest in any single harvesting expedition.

From the composite harvested insect sample, four samples (5 kg each) were prepared by transferring the bugs into clean zip lock bags. One fraction was frozen (Treatment A- clean method- unprocessed) (Fig 1c) and the other fraction, was killed by lukewarm water and heat dried (Treatment B: clean method- processed). Both treatments A and B were stored in the freezer (-80°C) until analysis for detection of aflatoxins.

#### Traditional preparation of insects

A sub-sample of the harvested insects (5 kg) was transferred into re-used grain bags and divided into two fractions (2.5 kg each). One of these fractions was kept in a traditional woven basket (Treatment C: traditional-unprocessed). The other fraction was killed using 5 L of lukewarm water (~37°C), then sieved and cooked by heating on a traditionally prepared clay pot. A colour change from green to golden brown which took approximately 3 min to develop indicated that the insects were well cooked. Dried insects were kept in traditional woven baskets (Treatment D: traditional-processed) at ambient temperatures until use for experiments (Fig 1a and 1b) Treatments A and B were used in the aflatoxin analyses only which showed negative results. Treatments C and D were used for aflatoxin, nutritional and secondary metabolite analyses.

### Chemicals

Aflatoxins B_1_, B_2,_ G_1_ and G_2_ were purchased from Supelco (Bellefonte, Pennsylvania, USA), apigenin (≥ 99%), luteolin (≥ 97%), rutin hydrate (≥ 94%) quercetin dehydrate (≥ 98%), octadecanoic acid (≥ 98.5%), glutamic acid, myristoleic acid, tetradecanoic acid, hexadecanoic acid, (*Z*)-9-hexadecenoic acid, linoleic acid, (*Z*)-9-octadecenoic acid, octadecanoic acid, (*Z*, *Z*)-9,12-octadecadienoic acid, oleic acid, eicosanoic acid and amino acid standard solution (AAS 18) were purchased from Sigma-Aldrich (Chemie GmbH, Munich, Germany).

### Analysis of mycotoxins

Mycotoxin analysis was carried out on traditionally processed and unprocessed stink bugs as well as samples collected in clean zip lock bags according to a modified method in reference [13]. The stink bugs (10 g) from each fraction were snap frozen in liquid nitrogen, crushed into fine powder and extracted in 40 mL acetonitrile-water (86: 16, v/v) for 30 min while sonicating. Each mixture was allowed to settle for 30 min and then 6 mL of each sample was filtered through a solid phase extraction (SPE) cartridge Multisep^®^228AflaPat multifunctional columns (Romer Labs, MO, USA). An aliquot (4 mL) of each cleaned extract was evaporated to dryness in a stream of nitrogen gas. The dried samples were re-dissolved in 400 μL methanol-water (20: 80, v/v), vortexed for 1 min and then centrifuged at 14, 000 rpm for 5 min prior to analysis using liquid chromatography coupled to quadruple time of flight mass spectrometry (LC-QtoF-MS). Samples derived from different harvesting, storage and processing procedures were analysed in five replicates.

### Analysis of fatty acids

Stink bug samples (traditionally processed and unprocessed) were snap frozen in liquid nitrogen, and then crushed into a fine powder. A methyl esterification reaction was then performed on 5 g of each sample according to a protocol adapted from [14]. A solution of sodium methoxide in methanol was prepared to give a concentration of 15 mg/mL. An aliquot of the soultion (500 μL) was added to each ground insect sample, vortexed for 1 min and then sonicated for 5 min. The reaction mixture was incubated at 60°C for 1 hr, thereafter quenched by adding 100 μL deionized water followed by vortexing for another 1 min. The resulting methyl esters were extracted using GC-grade hexane (Sigma–Aldrich, St. Louis, USA) and then centrifuged at 14, 000 rpm for 5 min. The supernatant was dried over anhydrous Na_2_SO_4_ and then analysed using gas chromatography coupled to mass spectrometry (GC/MS). Fatty acids were identified as their methyl esters by comparison of gas chromatographic retention times and fragmentation patterns with those of authentic standards and reference spectra publishedby library–MS databases: National Institute of Standards and Technology (NIST) 05, 08, and 11. Serial dilutions of the authentic standard octadecanoic acid (0.2–125 ng/μl) were analyzed by GC/MS in full scan mode to generate a linear calibration curve (peak area vs. concentration) with the following equation [*y = 7E+06x − 4E+07*(R^2^ = 0.9757)], which was used for the external quantification of the different fatty acids.

### Analyses of flavonoids

Three different samples; processed, unprocessed stink bugs and the leaves of *Vangueria apiculata*, the host plant from which stink bugs were harvested, were analysed for flavonoids. The samples were separately crushed into fine powder in liquid nitrogen. For each sample, 2.5 g were separately extracted in 50 mL methanol-water (80:20 v/v) by ultrasonication in a sonication bath (Branson 2510, Danbury, CT, USA) for 1 hr followed by filtration through a Whatman filter paper No. 32. The remaining residue was re-extracted twice, and the filtrate pooled separately. The extracting solvent was removed under reduced pressure at 40°C using a rotary evaporator (Laborata 4000; Heidolph Instruments GmbH & Co. KG, Germany) to give 60, 80 and 160 mg for unprocessed, processed and host plant leaves respectively. The extracts (5 mg) from each of the samples were re-dissolved in 1 mL water-methanol (95: 5 v/v), centrifuged at 14,000 rpm for 5 min and the supernatant analysed using LC-Qtof-MS. Five replicates were carried out with each replicate done on a different harvested and processed sample.

### Instrument conditions

#### LC-Qtof-Ms

The chromatographic separation was achieved on a Waters ACQUITY UPLC (ultra-performance liquid chromatography) I-class system (Waters Corporation, Milford, MA, USA). For amino acid analysis, the UPLC was fitted with an ACE C18 column (250 mm x 4.6 mm, 5μm (Aberdeen, Scotland) with a heater turned off and an autosampler tray cooled to 5°C. Mobile phases of water (A) and acetonitrile (B) each containing 0.01% formic acid was employed. The following gradient was used: 0 min, 5% B; 0–3 min, 5–30% B; 3–6 min, 30% B; 6–7.5 min, 30–80% B; 7.5–10.5 min, 80% B; 10.5–13.0, 80–100% B, 13–18 min, 100% B; 18–20 min, 100–5% B; 20–22 min, 5% B. The flow rate was held constant at 0.7 ml min^−1^. The injection volume was 1 μL.

For analyses of flavonoids and aflatoxin, a UPLC was fitted to a Waters ACQUITY UPLC BEH C18 column (2.1mm × 50 mm, 1.7 μm particle size; Waters Corporation, Dublin, Ireland) heated to 40°C and an auto sampler tray cooled to 15°C. Mobile phases of water (A) and methanol (B), each with 0.01% formic acid were employed. The following gradient was used for i) flavonoids: 0–0.2 min, 10% B; 0.2–3 min, 10–60% B; 3–5 min, 60–80% B; 6–8 min, 80% B; 8–9 min, 100% B; 9–10 min, 100% B; 10–10.5 min 100–10% B; 10.5–12 min 10% B, ii) aflatoxin: 0–0.2 min, 10% B; 0.2–3 min, 10–90% B; 3–5 min, 90% B; 5–6 min, 90–10% B; 6–7 min, 10% B. The flow rate was held constant at 0.4 ml min^−1^ for both analyses.

The UPLC system was interfaced with electrospray ionization (ESI) to a Waters Xevo QToF-MS operated in full scan MS^E^ in positive mode. Data were acquired in resolution mode over the *m/z* range 100–1200 for flavonoids and aflatoxin: *m/z* 100–700 for amino acid analysis with a scan time of 1 s using a capillary voltage of 0.5 kV, sampling cone voltage of 40 V, source temperature 100°C and desolvation temperature of 350°C. The nitrogen desolvation flow rate was 500 L/h. For the high-energy scan function, a collision energy ramp of 25–45 eV was applied in the T-wave collision cell using ultrahigh purity argon (≥99.999%) as the collision gas. A continuous lock spray reference compound (leucine enkephalin; [M+H] ^+^ = 556.2766) was sampled at 10 s intervals for centroid data mass correction. The mass spectrometer was calibrated across the 50–1,200 Da mass range using a 0.5 mM sodium formate solution prepared in 90:10 2-propanol/water (v/v).

MassLynx version 4.1 SCN 712 (Waters Corporation, Maple Street, MA) was used for data acquisition and processing. The elemental composition was generated for every analyte. Potential assignments were calculated using mono-isotopic masses with a tolerance of 10 ppm deviation and both odd- and even-electron states possible. The number and types of expected atoms was set as follows: carbon ≤ 100; hydrogen ≤ 100; oxygen ≤ 50; nitrogen ≤ 6; sulfur ≤ 6 [15]. The empirical formula generated was used to predict structures which were proposed based on the online database, fragmentation pattern, literature and confirmed using authentic standards.

Serial dilutions of authentic standards of aflatoxin B_1_ (0.01–20 ng/μl); rutin, quercetin, luteolin, apigenin (1.8–181 ng/μl); and glutamic acid (0.01–10 ng/μl) were also analyzed by LC-Qtof-MS in MS^E^ mode to generate linear calibration curves (peak area vs. concentration) with the following linear equations: aflatoxin B_1_ [*y = 13738x + 6611*.*5* (R^2^ = 0.9571)], rutin [*y = 5578*.*4x − 39094* (R^2^ = 0.9960)], quercetin [*y = 4372*.*4x + 79607* (R^2^ = 0.9854)], luteolin [*y = 13433x − 23256* (R^2^ = 0.9994)] apigenin [*y = 10288x − 11117* (R^2^ = 0.9995)] and glutamic acid [*y = 40137x − 1353*.*1* (R^2^ = 0.9999)] which served as the basis for the external quantification of the aflatoxin, flavonoids and amino acid.

#### GC/MS

Fatty acid methyl esters (FAMEs) were analyzed by GC/MS on a 7890A gas chromatograph (Agilent Technologies, Inc., Santa Clara, CA, USA) linked to a 5975 C mass selective detector (Agilent Technologies, Inc., Santa Clara, CA, USA) by using the following conditions: inlet temperature 270°C, transfer line temperature of 280°C, and column oven temperature programmed from 35 to 285°C with the initial temperature maintained for 5 min then 10°Cmin^−1^ to 280°C, held at this temperature for 20.4 min. The GC was fitted with a HP-5 MS low bleed capillary column (30 m × 0.25 mm i.d., 0.25 μm) (J&W, Folsom, CA, USA). Helium at a flow rate of 1.25 ml min^−1^ served as the carrier gas. The mass selective detector was maintained at ion source temperature of 230°C and a quadrapole temperature of 180°C. Electron impact (EI) mass spectra were obtained at the acceleration energy of 70 eV. A 1.0 μl aliquot of sample was injected in the splitless mode using an auto sampler 7683 (Agilent Technologies, Inc., Beijing, China). Fragment ions were analyzed over 40–550 *m/z* mass range in the full scan mode. The filament delay time was set at 3.3 min.

### Nutritional analysis

**Proximate analysis:** Crushed stink bugs were analysed based on procedures employed by the Association of Analytical Chemists [16]. The ash content was determined by heating at 550°C overnight. The organic matter (OM) was then determined by subtracting ash content from 100. Velp solvent extractor (SER 148/6) was used to determine fat content with ethyl ether as extractant. Crude protein (CP) was determined using the Kjeldahl method by determining the nitrogen content (%) and multiplying by the factor 6.25. The neutral detergent fibre (NDF) and acid detergent fibre (ADF) were analysed with the Velp fibre analyzer (FIWE 6) (VELP Scientifica, Usmate Velate, Italy) using reagents described [17].

**Amino acids:** The method for protein extraction was adopted from [18]. Processed and unprocessed insect samples were separately snap-frozen in liquid nitrogen and crushed into fine powder. The samples (2 g each) were extracted for 1hr in ice cold 5 v/w 100 mM 4-(2-hydroxyethyl)-1-piperazineethanesulfonic acid (HEPES) pH 7.2, 2 mM dithiothreitol (DTT), 2.5% Polyvinylpyrrolidone (PVP), 0.5 mM Ethylenediaminetetraacetic acid (EDTA), 1 mM benzamidine 0.1 mM phenylmethanesulfonylfluoride (PMSF) in a magnetic stirrer. The samples were filtered through KERLIX^™^ Gauze Bandage Rolls Sterile Soft Pouch 5.7 cm × 2.7 m centrifuged at 8000 rpm for 30 min at 4°C to remove solid debris. Protein was precipitated between 45% and 80% (NH_4_)_2_SO_4_ and the pellet recovered by centrifugation at 21,000 rpm for 30 min at 4°C. The protein pellets were desalted in 20 mM HEPES–NaOH pH 8 containing 2 mM DTT using Sephadex G-25 gel filtration chromatography (PD-10 columns, GE Healthcare) to give 80.2 mg and 77.9 mg of proteins from processed and unprocessed insect samples respectively.

10 mg from each of the samples were separately transferred into a 5 ml micro- reaction vial into which 2ml of 6N HCl were added and closed after careful introduction of nitrogen gas. The samples were hydrolyzed for 24 h at 110°C. For tryptophan analysis, 10 mg from each of the samples were separately transferred into a 5 ml micro-reaction vial into which 2 ml of 6N NaOH were added and then capped after careful introduction of nitrogen gas. The samples were hydrolyzed for 24 h at 110°C. After the hydrolysis, the mixtures were evaporated to dryness under vacuum. The hydrolysates were reconstituted in 1 ml 90:10 water: acetonitrile, vortexed for 30 s, sonicated for 30 min, and then centrifuged at 14,000 rpm and the supernatant analysed using LC-Qtof-MS. The analysis was replicated three times.

**Mineral analysis:** The crushed insects were ashed and digested in 6N HCl and the content of the various minerals were analysed on an Atomic Absorption Spectrophotometer (Model—AA6300, Shimadzu, Japan).

### Statistical analysis

Statistical analysis for ofaflatoxin, flavonoid and fatty acids contents and nutritional data were done using SAS statistical software [19]. The results were expressed as mean ± standard error. The means were separated using paired t-test for pair-wise comparisons and Tukey’s multiple comparison tests at P < 0.05.

## Results

### Analysis of aflatoxins

**Fig 2:**
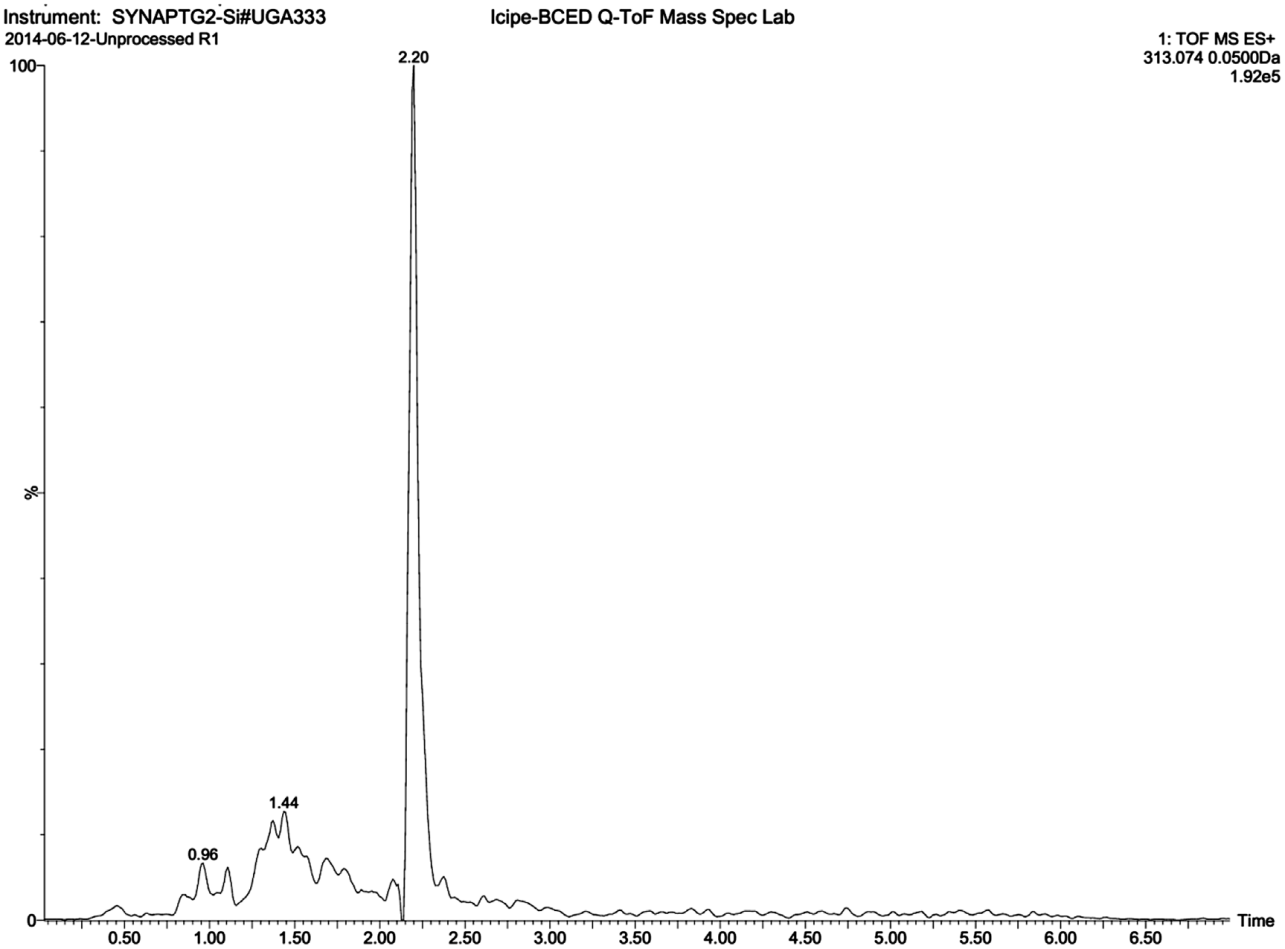
Representative Extracted ion chromatograms for *m/z* 313.0737. The retention time of aflatoxin B_1_ is 2.20 min.

LC-Qtof-MS analysis for mycotoxins identified only aflatoxin B_1_ in traditionally harvested unprocessed (0.50±0.17 ng/g) and processed (0.59±0.18 ng/g) insects (Fig 2). There were no significant (P>0.05) differences in the levels of aflatoxin B_1_ detected in the two different treatments. No aflatoxins were detected in insects that were harvested into and stored in clean zip lock bags.

### Fatty acid analysis

**Fig 3:**
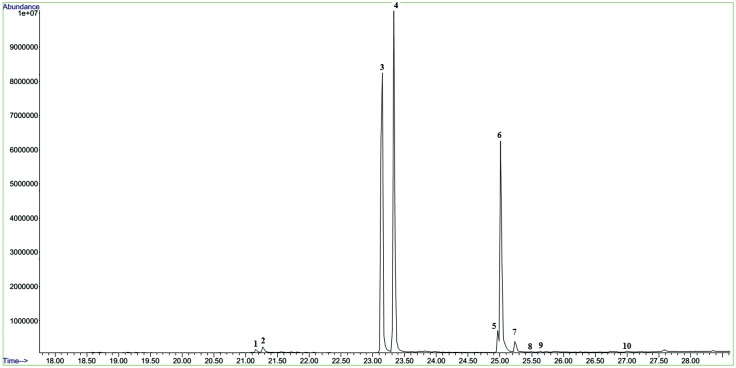
Representative total ion chromatogram showing fatty acid methyl esters (FAMEs) detected in processed and unprocessed *E*. *delegorguei*. Peaks 1–10 indicate FAMEs shown in Table 1.

A total of 10 fatty acid methyl esters (FAMEs) were identified from the unprocessed and the traditionally processed stink bug samples. Of these, seven were unsaturated fatty acid derivatives (methyl oleate, methyl (*Z*)-9-hexadecenoate, methyl linoleate, methyl (*Z*)-9-octadecenoate, methyl octadecanoate, methyl (*Z*,*Z*)-9,12-octadecadienoate and methyl myristoleate), while the remaining three were saturated fatty acid derivatives (Fig 3; Table 1).

**Table 1:** Concentration of fatty acid methyl esters (mg/g) in unprocessed and processed *E*. *delegorguei*.

Peak no	RT (min)	Fatty acid methyl esters	Unprocessed	Processed
1	21.17	Methyl myristoleate	0.40±0.05^(a)^	0.37±0.12^(a)^
2	21.29	Methyl tetradecanoate	0.6±0.17^(a)^	0.70±0.11^(b)^
3	23.15	Methyl (*Z*)-9-hexadecenoate	10.87±3.32^(b)^	10.17±3.42^(ab)^
4	23.35	Methyl hexadecanoate	12.53±3.75^(b)^	10.46±3.78^(b)^
5	24.96	Methyl linoleate	4.92±0.08^(ab)^	5.04±0.18^(ab)^
6	25.03	Methyl (*Z*)-9-octadecenoate	6.98±1.51^(ab)^	7.40±1.95^(ab)^
7	25.25	Methyl octadecanoate	5.22±0.64^(ab)^	5.05±0.17^(ab)^
8	25.47	Methyl (*Z*,*Z*)- 9,12-octadecadienoate	6.24±1.95^(ab)^	8.23±2.54^(ab)^
9	25.63	Methyl oleoate	0.36±0.07^(a)^	0.33±0.01^(a)^
10	27.00	Methyl eicosanoate	0.8±0.05^(a)^	0.37±0.02^(a)^

Retention time (RT). Concentrations of FAMEs bearing the same letter in either processed or unprocessed (along the column) are not significantly different (P≤0.05, Tukey’s HSD test).

The unsaturated fatty acids include some of the most essential fatty acids (linolenic acid and linoleic acid) for human nutrition. Except for eicosanoic acid, processing of the stink bugs did not significantly (P>0.05) affect the concentration of the fatty acids.

### Analysis of flavonoids

**Fig 4:**
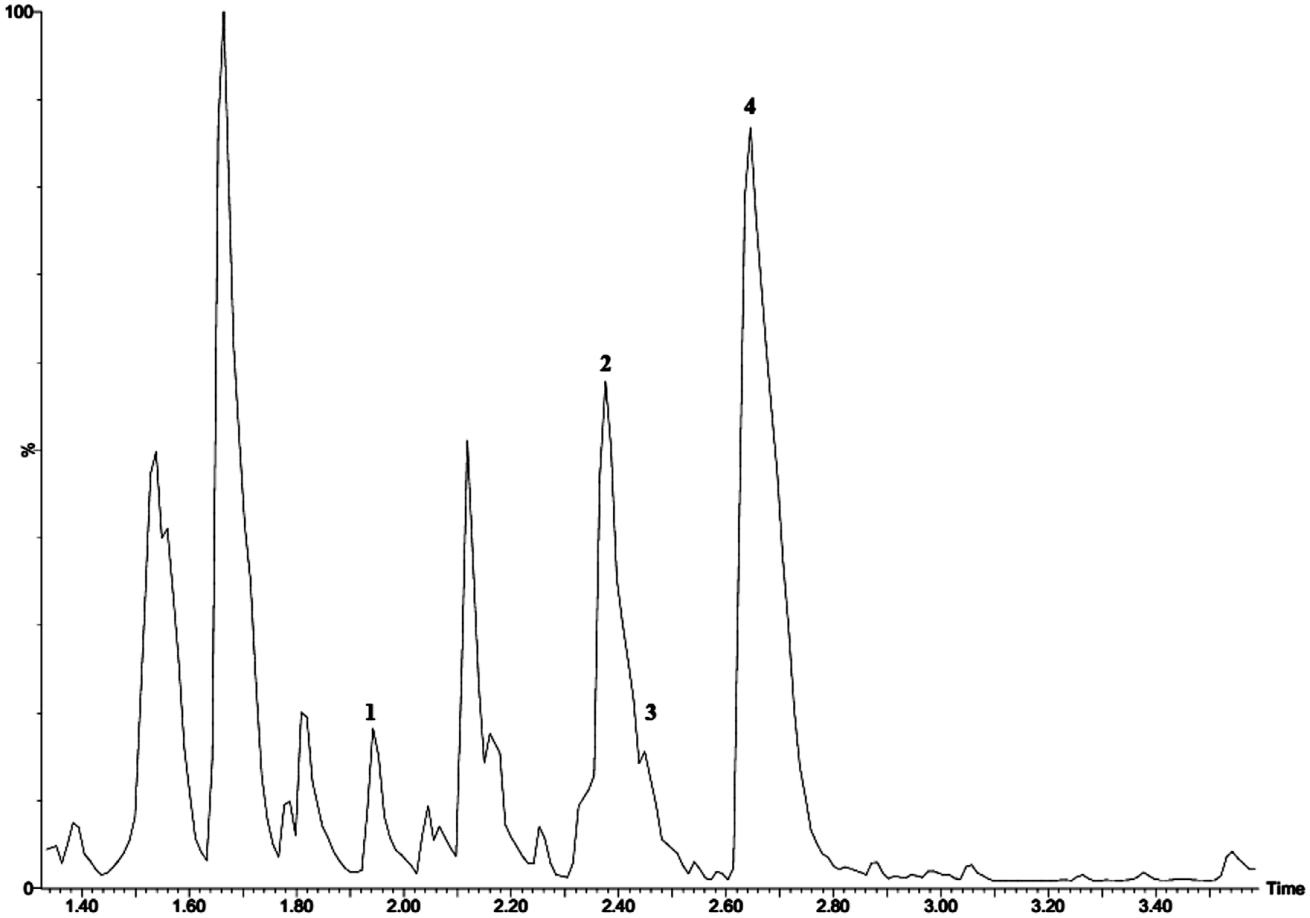
Representative total ion chromatogram showing flavonoids detected in samples of *V*. *apiculata* leaves and of *E*. *delegorguei*. Peaks 1–4 indicate the flavonoids shown in Table 2.

LC-Qtof-MS analysis showed the presence of four flavonoids in the three different treatments (unprocessed, processed and host plant *V*. *apiculata* leaves) (Fig 4; Table 2).

**Table 2:** Concentrations (ng/g) of flavonoids in unprocessed and processed *E*. *delegorguei* and *V*. *apiculata* leaves.

RT (min)	Flavanoid	Unprocessed	Processed	*V*. *apiculata* leaves
1.95	Rutin	2.89±0.54	2.89±0.43	75.76±21.53
2.39	Quercitin	4.33±1.57	4.18±1.15	231.02±61.55
2.46	Luteolin	3.25±1.05	3.29±0.81	146.48±41.36
2.66	Apigenin	37.13±12.59	34.52±10.02	1780.94±540.45

Generally, apeginin was the most abundant flavonoid while rutin was the least in all the samples analysed. All the four flavonoids were over 40-fold more abundant in the leaves than in the insect samples. Processing did not significantly (P>0.05) affect the flavonoid content.

### Nutritional Analyses

The proximate composition of stink bugs are presented in Table 3.

**Table 3:** Proximate composition (%DM) of unprocessed and processed *E*. *delegorguei*.

Parameter	Unprocessed	Processed	Significance (p<0.05)
Organic matter	98.4±0.08	98.2±0.07	**NS**
Crude protein	33.2±0.22	37.7±0.05	*
Fat	62.4±0.34	57.7±0.38	**
Neutral detergent fibre	37.3±0.78	32.6±1.13	*
Acid detergent fibre	17.5±0.18	19.0±0.20	*

*indicates significance at P<0.05

**indicates significance at P<0.01

^NS^indicates no significance at P>0.05

The processed stink bugs had significantly (P<0.05) higher levels of CP and ADF contents than the unprocessed ones. However, the unprocessed stink bugs had significantly (P<0.05) higher levels of fat and NDF than those that were processed.

The mineral content of the insects are presented in Table 4.

**Table 4:** Mineral content (% DM) of unprocessed and processed *E*. *delegorguei*.

Element	Unprocessed	Processed	Significance (p<0.05)
Zinc	0.02	0.01	*
Iron	0.07	0.08	**NS**
Copper	0.02	0.02	**NS**
Potassium	0.79	0.85	**NS**
Sodium	0.25	0.27	**NS**
Calcium	0.56	0.58	**NS**
Magnesium	0.16	0.17	**NS**
Phosphorus	1.39	1.46	*
Manganese	ND	ND	
Selenium	ND	ND	

*indicates significance at P<0.05

^NS^indicates no significance at P>0.05

^ND^indicates not detected

Most of the minerals analysed were detected in *E*. *delegorguei* except manganese and selenium. The mineral content was generally low except phosphorus. There were minimal differences in the levels of various minerals between processed and unprocessed stink bugs except for zinc and phosphorus.

**Fig 5:**
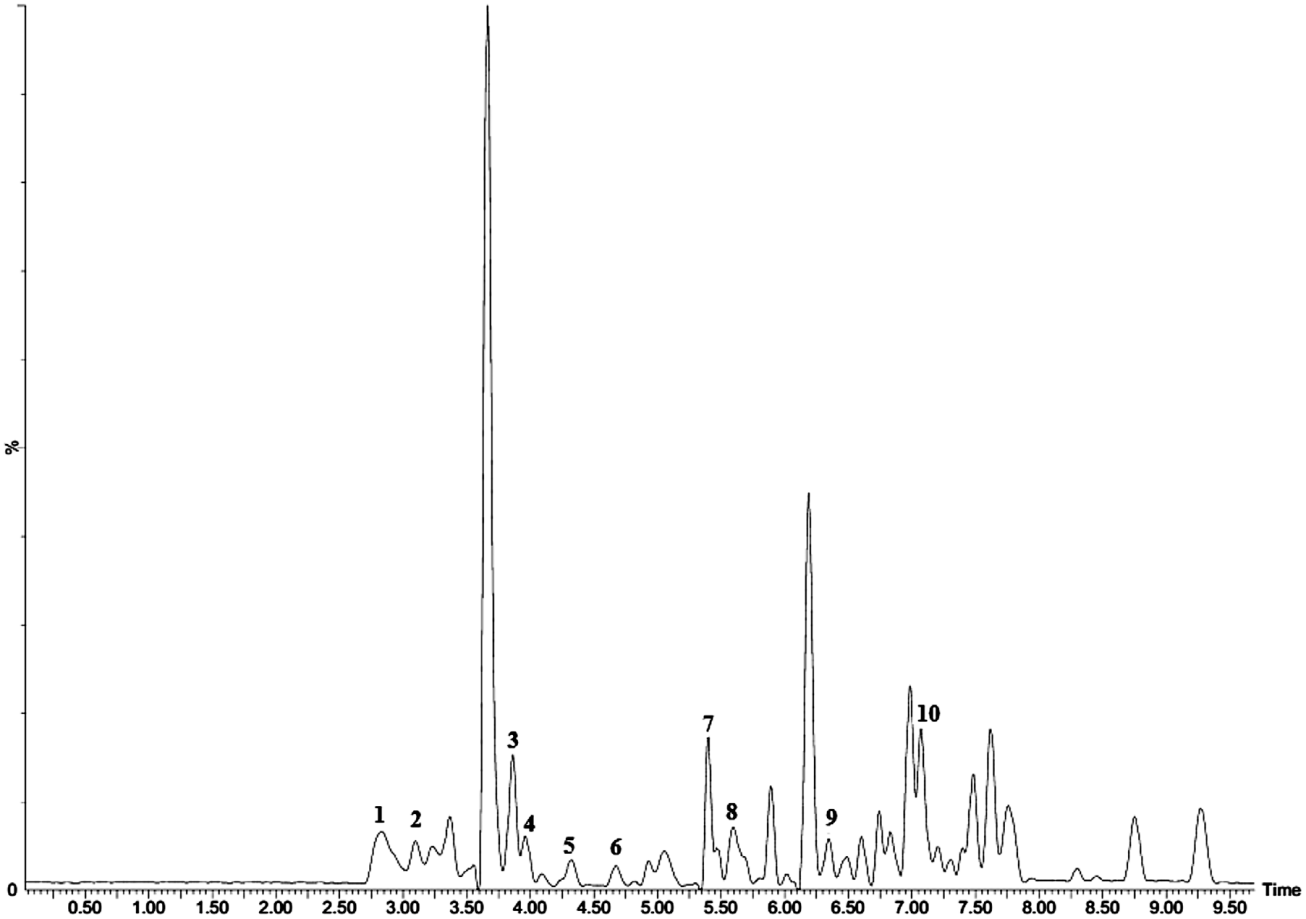
Representative total ion chromatogram of the amino acid profile of unprocessed and processed insects. Peaks 1–10 indicate amino acids shown in Table 5.

The amino acid profile of *E*. *delegorguei* is presented in Fig 5; Table 5.

**Table 5:** Concentrations (mg/g) of amino acids in unprocessed and processed *E*. *delegorguei*.

Amino acid	Unprocessed	Processed
Tryptophan	3.39±0.04	3.32±0.13
Arginine	3.03±0.06	3.16±0.08
Isoleucine	3.18±0.27	3.39±0.56
Leucine	7.11±0.18	7.44±0.71
Proline	2.44±0.16	2.42±0.14
Valine	0.97±0.17	0.98±0.15
Methionine	1.08±0.08	1.30±0.24
Hydroxyproline	2.57±0.08	3.06±0.07
Tyrosine	6.37±0.10	6.97±0.41
Threonine	0.42±0.02	0.55±0.08
Lysine	0.88±0.02	0.86±0.05
Phenylalanine	2.10±0.06	2.37±0.09

Nine essential amino acids were detected including the most limiting (lysine, tryptophan and methionine) in cereal/legume based diets. Three non-essential amino acids were also detected. The concentrations of the amino acids ranged from 0.86 mg/g (lysine)– 7.44 mg/g (leucine) for processed and 0.88 mg/g (lysine)– 7.11 mg/g (leucine) for unprocessed insects. However, processing did not significantly (P>0.05) affect the amino acid content.

Tryptophan was identified in both the processed and unprocessed stink bug based on accurate mass determination and literature data as follows: The LC-Qtof-MS peak at retention time 2.33 min had a molecular ion peak [M+H]^+^ at *m/*z 205.0974 and fragment ions *m/z* 91.0557, 118.0658, 132.07427, 146.0623 and 188.0244, elemental composition of C_11_H_13_N_2_O_2_, 1.5 ppm error to theoretical value and a fit confidence of 99.9% consistent with tryptophan [20].

## Discussion

Aflatoxins are a group of toxic compounds belonging to the difuranocoumarins and produced by *Aspergillus flavus* and *Aspergillus parasiticus* [21] in/on cereals, oils seeds, spices, chillies and milk [22]. Growth and development of fungi producing these toxins is favoured by warm temperatures and high humidity [22]. The presence of aflatoxins in human and animal food is undesirable. In humans, a condition called aflatoxicosis, is a primary hepatic disease associated with aflatoxin B_1_ [23].

In carrying out our study, we observed and hypothesised that traditional harvesting and storage practices of *E*. *delegorguei* favour the occurrence of mycotoxins as polypropylene bags and wooden baskets are alternatively used to store cereal grain and legumes which are often associated with mycotoxin contamination [23]. This hypothesis was proven correct by absence of aflatoxins in insects collected and handled using clean and non-contaminated bags.

Our study confirmed the occurrence of aflatoxin B_1_ (AFB_1_) in traditionally collected and stored processed and unprocessed insect samples. AFB_1_ is one of the most potent naturally occurring mutagens and carcinogens which cause significant threats to the food industry and animal production [23]. Although the levels detected in the insect sample (0.50 ng/g and 0.59 ng/g for unprocessed and processed samples respectively) were below the maximum limits recommendations (20 ng/g;World Health Organization), concern still remains for the possible adverse effects resulting from long-term exposure to low levels of aflatoxins in the use of the edible stink bug as food for human consumption.

Our study was able to associate aflatoxin contamination of edible stink bugs with traditional harvesting and stored practices of insects into wooden baskets and old polypropylene grain bags. However, a follow up study could involve assessing the levels of contamination of these containers before using them for harvesting and storage of edible stink bugs. The nature of the wooden baskets with interwoven strings smeared with cow dung can potentially result in surface moisture retention which can promote growth of moulds. On the other hand, harvested and processed insects (usually with moisture content of 33–45%) can get cross-contaminated by the toxin causing fungi or their spores from storage in the old polypropylene bags. In general, environmental humidity of 70–90%, storage temperature of 20 to 40°C and substrate humidity of 10–20% are known to favour aflatoxins production [24]. Therefore, using the current harvesting and traditional handling and storage practices in the South-eastern districts of Zimbabwe poses a great risk to consumers from mycotoxins contamination. However, use of alternative clean plastic bags demonstrated that the risk can be reduced/eliminated.

Analyses of fatty acids revealed in general a composition of mostly unsaturated fatty acids (seven out of detected ten fatty acids) in processed and unprocessed insects. Unsaturated fatty acids play important functions to insects such as regulating fluidity of membranes and as pre-cursors of many pathways for the synthesis of substances such as waxes, pheromones, eicosanoids and other defense secretions [24]. For humans, unsaturated fatty acids are generally considered healthy because they help in reducing cholesterol levels which is often associated with heart diseases [25]. Specifically the detection of methyl palmetoleate and methyl oleate which confirmed the presence of the monounsaturated fatty acids palmetoic and oleic acids in *E*. *delegorguei* is of great benefit to consumers. Studies have shown that these two fatty acids or their derivatives promote cell viability and mitogenesis and they help to protect β-cells from glucose and palmatic acid-induced apoptosis [26, 27]. On the other hand, the saturated fatty acids palmitic acid and stearic acid identified in this study have been shown to enhance flavour and having hypocholesterolemic effects [28].

Linoleic acid was the only essential polyunsaturated fatty acid found in unprocessed and traditionally processed stink bugs. The edible stink bug therefore is an important source of this vital fatty acid which has to be obtained from dietary sources. Several health and nutritional benefits of this fatty acid in diet include hypocholesterolemic [29], anticoronary [30] and antiarthritic [31]. Linoleic acid identified in the edible stink bug is expected to lessen deficiencies to consumers in communities where vegetable and animal sources may be limited.

Four flavonoids including rutin, quercitin, luteolin and apeginin, were detected in unprocessed and processed stink bugs in similar amounts, and these metabolites were traced back to their feed source *V*. *apiculata* leaves, which contained relatively high levels of these compounds. Flavonoids influence selection of host plants by the phytophagous insects such as the edible stink bug because they may serve a defence function in insects against natural enemies [32,33]. It is most likely that the insects acquired these compounds during herbivory by the actively feeding nymphs which then stored/ sequestered them throughout to the adult diapausing stages. This suggestion however requires further investigation. The four flavonoids reported here are all aglycones found in many plants [33,34]. The edible stink bug would therefore, be a source of these flavonoids, which are normally found in vegetables and fruits in human diets. Studies have shown that these compounds have health-promoting properties due to their high anti-oxidant capacity [35]. Overall, flavonoids have so far been found to exhibit a wide spectrum of pharmacological properties, including antioxidative, antiallergic, antiinflammatory, antidiabetic, hepato- and gastro-protective, antiviral, and antineoplastic activities [35,36].

Insects contain proteins where they perform various structural and physiological roles vital for their survival. Edible insects have been shown to have higher protein content, on a mass basis, than other animal and plant foods such as beef, chicken, fish, soybeans, and maize [10]. Ramos [37] reported the nutritional value of 78 species of edible insects in Mexico, with protein values ranging from 15–81%, and calorie content ranging from 293–762 kcal/100 g. The protein content (37.7% and 33.2%) in the current study was similar to that reported from South Africa (35.2%) [10]. Teffo et al. [38] suggested that processing of the insects would have an effect on the nutritional quality of the insects. Our study also found significant effect on the nutritional quality of processing the edible stink bug. The crude protein was increased with processing while the fat content also significantly reduced. The increase in crude protein content is because of the reduction in water content due to cooking which concentrates the nutrients. However, during the processing of the bugs, some of the fats appeared to have been lost due to heating.

The fat content in insects is an indication of the amount of energy reserves found in the insect body and has an inverse relationship with the length of the diapausing period [9]. The fat content of *E*. *delegorguei* was higher than the values previously reported [10]. These differences could be due to the different analytical and sensitivity of the methods used in the two studies. Our method was according to Randall technique (using hot solvent) while [10] used the Soxhlet technique (using cold solvent). Fats are essential in daily human diets as they increase the palatability of foods by absorbing and retaining their flavours [39]. They are also vital in the structural and biological functioning of cells and help in the transport of nutritionally essential fat-soluble vitamins [40]. The fat content of *E*. *delegorguei* therefore represents a good source of energy. This result is consistent with the results of [10] for *E*. *delegorguei* and for other edible insects [41].

The organic matter of the *E*. *delegorguei* was very high (98.2% and 98.4%), which implies low ash content. The low ash content was also reported [10] for *E*. *delegorguei*, which is consistent with the results of this investigation. The low ash content of *E*. *delegorguei* implies low mineral content. This was confirmed by low contents of the various minerals analysed. However, *E*. *delegorguei* will supply appreciable quantities of phosphorus. The mineral content obtained in this study was lower than that reported [10]. Mineral content in organisms vary depending on the location and type of feed consumed by the organism. The fibre content of insects is mainly influenced by the insect exoskeleton and structure mainly chitin. The fibre content of the *E*. *delegorguei* was moderate. The consumption of fibre helps in digestion of foods by enhancing peristaltic movements of the digestive system as well as providing bulk to food and therefore gives a sense of satiety even when small amount of food is eaten [41].

The edible stink bug has both essential and non-essential amino acids. While [10] reported 8 essential amino acids, our investigation revealed 9 essential amino acids, which indicates the high quality of proteins from *E*. *delegorguei*. All the three most limiting amino acids in plant based diets (lysine, methionine and tryptophan) were detected. This shows the importance of *E*. *delegorguei* as a source of high quality proteins to supplement plant based diets which is the common scenario in the developing countries. The values of the various amino acids in our study were lower than the values reported by [10]. This variation could be to due differences in the hostplants fed upon by the bug before collection, which in turn might be influenced by the season and environmental conditions at the sites in the two studies. Since there is scarcity of information on the influence of host plants and environmental conditions on the nutritional composition of edible insects, more studies are needed to address this gap.

## Conclusions

Aflatoxin B_1_ was found in both traditionally collected and stored unprocessed and processed insect samples but was not detected in samples harvested in clean zip-lock bags. Proper storage and handling of *Encosternum delegorguei* which is a good food source for human consumption are therefore important to avoid introducing aflatoxin contamination. Use of alternative cheap materials such as plastic–lined gunny bags which are easy to clean would help to reduce aflatoxin contamination. The presence of flavonoids which were also detected in the host plant would confer health benefits of flavonoids to humans upon consumption. We conclude that the edible stink bug is a nutrient- and antioxidant- rich food source for humans especially in cereal based diets since it contains high amounts of proteins, fats and phosphorus. The fats also contain higher proportions of unsaturated than the saturated fatty acids.
